# Linking individuals on probation to health care: a pilot randomized trial

**DOI:** 10.1186/s40352-020-00110-w

**Published:** 2020-03-31

**Authors:** Daniel J. O’Connell, Christy A. Visher, Patricia Becker

**Affiliations:** Center for Drug & Health Studies, Department of Sociology and Criminal Justice, University of Delaware, Newark, DE USA

**Keywords:** Probation, Health care, NIATx, Change team

## Abstract

**Background:**

Probation offices represent a location where at-risk individuals in need of health care appear on a known and regular basis. We sought to study how providing linkages to health care could improve the proportion of underserved, justice-involved individuals accessing the health care system. This study tested a linkage and referral to health care intervention for individuals on probation designed by a local change team that brought together actors from multiple agencies and tasked them with increasing general practitioner physician access for probationers. The pilot trial randomized 400 individuals on probation in Delaware during 2016–2018 to determine the effectiveness of placing a health navigator in an urban probation office to refer people to an appointment with a primary care physician. The project also tested the impact of offering an incentive to probationers for attending a doctor’s appointment.

**Results:**

Referral by a health navigator to a primary care physician was associated with a modest but significant increase in the proportion of individuals accessing care through a general practitioner physician. Offering an incentive had no significant impact on keeping the medical appointment above the effect of referral by the health navigator.

**Conclusions:**

Probation offices represent a location where at-risk individuals in need of health care appear on a known and regular basis. This study highlights how providing linkages to health care can improve the proportion of underserved individuals accessing the health care system.

## Background

Research has documented that people involved in the criminal justice system carry a disproportionately higher amount of the United States health care burden (Davis & Pacchiana, 2004). Studies of jail and prison inmates have demonstrated heightened risk of chronic diseases such as hypertension, asthma, and cervical cancer among prison inmates, even after controlling for known confounders such as age (Binswanger, Redmond, Steiner, & Hicks, 2011). Moreover, the prevalence of infectious diseases among incarcerated populations well surpasses that of non-incarcerated populations (Taxman & Ressler, 2010). In addition to these significant physical health disparities, rates of co-occurring disorders including behavioral health problems (e.g., substance misuse and mental health conditions) are alarming (Semenza & Grosholz, 2019).

While great attention has been paid to the fact that over 2 million individuals are incarcerated in U.S. prisons or jails, much less attention is given to the fact that there are nearly 4.5 million people under community supervision (Kaeble & Cowhig, 2018). The heightened risks of chronic, infectious and co-occurring health problems that incarcerated individuals endure is pertinent to this study since most of the individuals who are incarcerated in the United States will be released from custody and reenter the community under probationary supervision (Hughes & Wilson, 2002). The prevalence of infectious disease, as well as substance use and mental illness in the justice-involved population, has important implications for individual and community health. For example, treatment disruption for certain illnesses and diseases can cause additional health problems. Specifically, HIV treatment interruptions can lead to added health problems, particularly a greater chance of contracting other infectious diseases, such as pneumonia (Baillargeon et al., 2010). Subsequently, those implications impact the health of the communities to which they are returning, since the prevalence of infectious disease is returning with them.

A national survey (Vaughn, DeLisi, Perron, & Abdon, 2012) sheds light on how those under correctional supervision have disproportionately higher rates of mental illness, drug addiction, and other health problems. Results from this survey found that probationers, in comparison to those not on probation, reported higher rates for several health-related conditions: anxiety, 1.6 times; depression, 1.8 times; asthma, 1.5 times; sexually transmitted infections, 3 times. The same study indicated that probationers were between 3 and 7 times more likely to have symptoms of substance use disorders, 12 times more likely to report past drug or alcohol treatment exposure, and 3 times more likely to report receiving mental health treatment in their lifetime, as compared to those not on probation.

Similarly, Visher and Mallik-Kane (2007) analyzed longitudinal data to understand the physical and mental health conditions of those incarcerated and the proportion of those who were linked to health care upon release. They found that over four out of five former prisoners had at least one of these conditions and two out of five had multiple conditions. Moreover, access to care after release did not match access to care while incarcerated. Roughly three in five men with physical or mental health conditions received treatment in prison, whereas 60 days after release approximately two in five received treatment. Furthermore, about one-half of men with a history of substance abuse participated in recovery programs (including AA or NA) in prison, whereas less than one-third participated 2 months after release.

Research on the health needs of probationers is scarce compared to that of incarcerated persons. Individuals on probation experience higher rates of physical health conditions, substance use, and mental illnesses because of several factors. It appears that the same reasons that increase the likelihood of criminal justice involvement are also the reasons that impact the health status of these individuals. That is, individuals on probation are primarily from disadvantaged neighborhoods and are disproportionately male, Black, poor, and undereducated (Hawkins, O'Keefe, & James, 2010). In fact, an estimated 33% of Black men will be under correctional supervision sometime in their life, in contrast to 17% of Hispanic men and 6% of white men (Bonczar & Beck, 1997). These racial disparities are important because scholars have shown that racial and ethnic minority groups, including Blacks, Hispanics, and American Indians, receive poorer health care and have worse health outcomes than non-Hispanic whites (James, Thomas, Lillie-Blanton, & Garfield, 2007). Additionally, racial and ethnic minorities are more likely to reside in low income communities where providers are less likely than other physicians to be board certified and less able to provide high quality care and referrals to specialty care (Bach, Pham, Schrag, Tate, & Hargraves, 2004).

Increasing numbers of individuals are being released into their home communities under probationary supervision, where they may not have access to regular health care and more typically utilize emergency health services (Pew Center on the States, 2010). The health status of probationers is particularly important now that states are attempting to reduce budgets through utilizing probation and similar community-based options, rather than incarceration. Individuals that come into contact with the criminal justice system and are placed on probation typically come from a socially disadvantaged background; therefore, effective health care must include broader environment and social determinants of health, such as housing, income, and family supports.

Many incarcerated individuals, particularly those who lived in impoverished conditions prior to incarceration, experience better health and receive better health care inside the correctional facility than they did in the community (Massoglia & Schnittker, 2009; Springer & Altice, 2005). Although health care may be below average in many correctional facilities (Greifinger, 2010; Springer & Altice, 2005), the regulated structure of correctional settings essentially affords a temporary reprieve from the otherwise hectic lifestyles of many incarcerated individuals that frequently include homelessness, substance abuse, and mental health disorder (Springer & Altice, 2005). Incarcerated individuals are provided with a sheltered place to sleep, three meals a day, and usually some degree of education, exercise, and physical and mental healthcare (Massoglia & Schnittker, 2009). The confined structure of correctional settings provides a context in which health care providers work in the facility and provide care in a structured manner, as opposed to individuals having to initiate treatment themselves and navigate the health care system in the community (Meyer, Chen, & Springer, 2011).

Yet, individuals on probation are often not afforded this same structured access to health care. Prior to the passage of the Affordable Care Act (ACA), single men with low incomes, such as many probationers, were generally not able to access Medicaid. In one study of men recently released from incarceration prior to ACA, 68% indicated that they had no health insurance (Visher & Mallik-Kane, 2007). After adoption of ACA, many states expanded Medicaid access to single men with limited incomes. This change enabled justice-involved men to access health care in the community. However, even with enrollment and care coordination efforts in place, many gaps and challenges remain in connecting them to care. Amidst the maze of barriers facing those on probation, caring for fundamental health concerns often pales in importance as they navigate an environment filled with urgent needs, such as housing and employment.

Although several studies have examined the health status of justice-involved individuals (Davis et al., 2009; Haney, 2017; James & Glaze, 2006; Mumola & Karberg, 2006; Wilper et al., 2009), few studies have attempted to implement a linkage to care process for individuals on probation. This article reports on the Delaware Culture of Health pilot study, which sought to capitalize on the fact that a group of persons with high health care risk and unmet health care needs gather on a known and regular basis at urban probation departments.

The overall goal of the Delaware Culture of Health pilot study was to determine whether probationers could be linked to general practice family doctors to decrease their reliance on emergency room visits and increase their utilization of routine health screening. Only the initial linkage to care outcome was targeted in this pilot study. Thus, the study tested the following hypothesis:

Screening and referral of probationers by an on-site health navigator will lead to a greater proportion of probationers accessing health care services compared to those receiving an educational health care workbook.

The project had five overlapping goals relating to the main goal: 1) assemble a team of practitioners from multiple agencies; 2) utilize a process improvement model based on the Network for Improvement of Addiction Treatment (NIATx) change team approach (McCarty et al., 2007) to facilitate a patient-focused view of health care navigation and patient need and design a mechanism to link persons on probation to primary care doctors; 3) develop a “Healthier You” educational workbook and self-improvement guide, 4) implement and test, using a randomized controlled trial research design, whether an interactive linkage to care process in an urban probation office would outperform a passive, information centered approach in linking persons to health care, and; 5) disseminate results from the study to researchers and practitioners in the field. As discussed below, an incentive was later added to the study design to improve the likelihood of participants appearing for their health care appointment after initial referral.

## Methods

The Center for Drug and Health Studies at the University of Delaware implemented a pilot study to investigate the process and short-term impact of implementing a multiagency “Culture of Health” team to develop an intervention that would link persons on probation to health care. The project worked by bringing together practitioners from state and community agencies and utilized a “change team” approach based on the NIATx process improvement model. Change teams utilize and rely upon the expertise of persons involved in the day to day operations of agencies to improve agency functioning or achieve a particular goal. Research staff used their contacts in multiple state and community agencies to bring the change team together, provide them with the overall goal of increasing probationer utilization of primary care physicians, and empower them to seek a means of reaching the goal by using their expertise with the justice and healthcare systems and their clients. The researchers then relied on the change team to determine the mechanism of getting probationers to health care. A more detailed description of the Culture of Health pilot study and its implementation approach is available elsewhere (Becker, O’Connell, & Visher, 2021).

All parties agreed in early change team meetings that the probation office represented a convenient place to access an underserved and at-risk population. All parties were equally adamant that, while the probation office was uniquely suited to access the population, health care screening and referrals should not be attached to the actual judicial probationary process. Probation administrators gave the Culture of Health team access to office space and allowed the team to utilize the probation waiting room to recruit respondents. All parties were clear that probation officers could not mandate that probationers see or interact with the team.

The project eventually placed a health navigator, who was a member of the research team with experience working with both probationers and in medical environments, on site at the probation office to refer people to health care appointments. A “Healthier You” workbook that contained information about good health practices, including the need for regular checkups and screening, as well as local information about how to contact and make appointments with doctors, social service agencies, and community treatment providers was developed for the study control condition. In addition, large gender-specific, health-related posters emphasizing the need for regular screenings were placed in the probation waiting room. Finally, a series of public service health videos were compiled and interlaced with local community members speaking of the need for regular health care visits, and were displayed on a large monitor in the waiting room. The idea was to create an environment in the waiting room where people were encouraged to think about health care. The project was initiated in the summer of 2017.

The pilot study randomized 400 probationers to either meeting with the health navigator or receiving the Healthier You workbook. Probationers were informed of the study by the research team who made periodic in person announcements in the probation office waiting room. Research staff informed individuals of the study, which included a $10 incentive in the form of a gift card for filling out a brief survey, and asked for volunteers. Interested persons were taken to a private screening area within the probation office, provided informed consent, and given the survey. They were then randomized to either the Culture of Health condition, which meant meeting with the health navigator, or the control condition, which was receipt of the Healthier You workbook. Individuals in the control condition were given the workbook and urged to utilize it and to make an appointment with a healthcare provider if they did not have a doctor.

Those randomized to the Culture of Health condition met with the health navigator, who explained the need for regular screenings and the benefit of have a regular doctor (e.g. personal relationship, avoiding long delays and costs at the ER, etc.). The health navigator then offered to make a doctor’s appointment for the participant at a location of their choosing. Those who agreed were given an appointment through a local health network; on average, appointments were within 2 weeks of the referral. Data were then collected from the health provider at regular intervals to determine who had attended these appointments.

Consent procedures for both conditions included allowing the research team to query the main local health provider to determine whether people in both conditions attended an appointment with a health care provider within 90 days. Outcome data were derived from electronic health records from the main health network in the study area. Randomization was conducted through an urn program balanced on gender, race, age, and probationers who had served prison terms preceding probation versus those sentenced to probation with no prison term.

## Results

There were no significant differences between the two randomized groups on any of the urn variables. Randomization achieved reasonable balance in the covariates. Table 1 displays descriptive characteristics for the sample broken down by treatment and control groups. As Table 1 shows, the study sample was approximately 73% male, which is slightly lower than the probation population which is 77% male. White respondents comprised 31.9% of the control group and 35.8% of the treatment group. While the study sample was not reflective of the overall probation population by race which is 51% white, this difference did not carry across treatment groups.

**Table 1:** Descriptive Statistics (*n* = 400)

	Control Group	Treatment Group
*Gender*		
Male	73.4%	72.6%
Female	26.1%	27.4%
Other	.5%	0%
*Race*		
White	31.9%	35.8%
Black	56.9%	50.8%
Asian	0%	.5%
American Indian	.5%	1.6%
Pacific Islander/Native Hawaiian	.5%	.5%
More than one race	4.4%	3.2%
Other	5.9%	7.5%

Table 2 displays results of survey data on prior health care utilization. Data collected as part of the study showed that the majority of participants had some form of health insurance. For participants in the control group, 69.8% reported having Medicaid and approximately 11% had insurance either through their family (5%) or employer (5.9%). Similarly, the majority of participants in the treatment group reported having Medicaid (67.9%) and nearly 10% reported having insurance through their employer (4.7%) or a family member (4.2%). Regardless of group assignment, over half of participants reported not having a primary care doctor (51.9% control; 59.2% treatment). Qualitative interviews indicated that many of those reporting having a doctor did not see them regularly. Somewhat paradoxically, 48.5% of the control group and 53.9% of the treatment group reported that their usual source of medical care was a primary care doctor. However, many indicated in interviews that they had not seen their doctor in years. Alarmingly, 30.6% of participants in the control group and 25% of participants in the treatment group reported using the emergency room as their primary source of medical care.

**Table 2:** Prior Health Care Utilization

	Control Group	Treatment Group
*Health Insurance Coverage*		
Yes, through work	5.9%	4.7%
Yes, through family	5%	4.2%
Yes, Medicaid	69.8%	67.9%
Yes, Other	2.5%	3.2%
None	16.8%	20%
*Have a Primary Care Doctor*		
Yes	48.1%	40.8%
No	51.9%	59.2%
*Usual Source of Medical Care*		
Primary Care Doctor	48.5%	53.9%
Clinics	6.6%	8.3%
VA	1.5%	1.7%
Urgent Care	2.6%	2.8%
Emergency Room	30.6%	25%
None (self-care)	7.7%	5.6%
Other	2.6%	2.8%

The main outcome was whether participants presented for a doctor’s appointment within 90 days of referral. Following standard practice for randomized trials and using an intent to treat approach, we present bivariate cross tabulation analyses and effect sizes in Table 3.

**Table 3:** Overall Study Outcomes: Percent Attended Doctor Appointment for Full and Reduced Samples

	% Attended Doctor Appt. Full Sample (95% CI) (*n* = 400)	Std. Dev.	% Attended Doctor Appt. Reduced Sample (95% CI) (*n* = 223)	Std. Dev.
Workbook Condition	16.9% (11.8–22.1%)	.376	10.1% (4.1–16.1%)	.302
Culture of Health Condition	23.4% (17.4–29.5%)	.424	25.6% (15.7–35.6%)	.441
Mean Difference	6.5%* (1.4–14.4%)		15.5%** (4.5–26.5%)	
Between Group Difference Effect Size	.33		.42	

* *p* < .10, **p < .01

Eighty people in the study sample of 400 persons (20%) attended a doctor’s appointment within 90 days of study enrollment. Examining the data by condition, 45 persons (23.4%) in the treatment condition) attended a doctor’s appointment while 35 persons (16.9%) in the control (workbook) condition attended a doctor’s appointment. The Pearson chi-square test *p*-value was .09.

Results from the baseline survey indicated that 223 (55.9%) of the probationers screened did not have a primary care physician. When the data were reanalyzed by selecting only the 223 individuals who did not have a doctor and thus could be considered eligible for referral to a primary care physician (reduced sample in Table 3), 20 persons (26%) in the treatment condition attended a doctor’s appointment while only 10 (10%) persons in the control condition did so. This difference was significant at *p* < .01. The overall full sample effect size was .33 indicating a modest effect. Results with the reduced sample examining only the 223 persons who did not have a regular doctor showed a moderate effect size of .42.

It should be noted that after early results indicated few people were appearing for their scheduled doctors’ appointments, change team members suggested offering a small incentive to those who presented at an appointment. The study was thus modified to offer a $20 gift card to those who attended a doctor’s appointment. Participants were thus randomized into one of the two main study conditions, and then each arm was randomized to either be offered the incentive or not. Contrary to expectations, offering the incentive had no significant impact on the likelihood of attending a doctor’s appointment (results not shown).

## Discussion

The major impact of this project was linking individuals on probation to primary care doctors: overall, 80 of the sample of 403 persons (20%) attended a doctor’s appointment. Second, 45 persons (23%) in the treatment condition (saw the health navigator) attended a doctor’s appointment while 35 persons (17%) in the control (workbook) condition attended a doctor’s appointment (effect size = .33). When selecting only those 223 people who did not have a doctor and thus were truly eligible for referral, 20 persons (26%) in the treatment condition attended a doctor’s appointment while only 10 (10%) of persons in the control condition did so (effect size = .42). These results show that the pilot study succeeded in linking individuals to health care resources. Furthermore, by creating the Culture of Health workbook, this project not only directly linked individuals to health care resources but also increased access to health care information.

The Delaware Culture of Health pilot study grew from the idea that a large at-risk and underserved population gathered at known and regular times in a public place: large urban probation departments. The project thus sought to determine whether actors in systems that generally did not interact, criminal justice agencies and large health care providers, could join together to increase access to health care for this high need and underserved population. The project demonstrated a number of lessons. First, correctional agencies, specifically a probation department, are both willing and able to coordinate with community health agencies to provide access to health services for their populations. Likewise, community health agencies were willing to bridge systems to increase access for an underserved, high need population. While a correctional agency may not have a primary responsibility to refer probationers to health care, the study demonstrated that they were more than willing to cooperate with multiple agencies to provide access to care for probationers. Second, health care organizations and state health agencies were willing to meet and to coordinate with other entities in the Delaware Culture of Health change team to design the screening and referral model utilized in the pilot study. The project thus bridged multiple systems in an effort to increase access to care for probationers. Lastly, the pilot study demonstrated proof of concept that placing a health navigator in a probation office to conduct screening and referrals significantly increased the likelihood of a probationer attending a healthcare appointment.

Considering the cost of placing hired staff in a probation office to refer probationers to health care, it is also worth examining the study’s effects in the control condition. About one in six individuals in the control condition (17.5%; 35 of 200) who were given a resource guide made and attended an appointment with a doctor. These results indicate that while an on-site health navigator is preferred, it may be enough to equip individuals on probation with the health resources needed to make appointments in order for some to engage with a health care provider. These findings indicate that this justice-involved population did exercise personal agency. This is to say that some individuals did not necessarily need someone to make an appointment for them; they just needed the resources to do so.

There are several limitations to this pilot study. First, the State of Delaware has a unified correctional system with all adult justice system operations (I.e., jail, prison, community supervision) under central management. Other states operate differently which could affect their ability to implement a similar intervention. Second, Delaware is a small state so we were able to build the relationships needed for a change team. Larger states may have difficulty implementing a similar change team process. Third, the study only collected data at one urban probation office. Fourth, we worked with the largest provider of health care in the state for client referrals and data on appearances for appointments, but the lack of access to state Medicare data restricted us from getting a full picture of doctor’s appointment visits outside of this primary health care provider.

## Conclusion

This pilot study demonstrates the potential of utilizing community corrections environments to link underserved persons to health care. The results indicate that with minimal guidance, a modest proportion of this population can be linked to care. There are 4.5 million persons under correctional care in the United States. If, as this study shows, approximately half of those do not have a regular doctor and efforts such as this one could link one in five of them to health care, there is a potential to improve the health of over one million people annually. The Delaware Culture of Health pilot study was the first of its kind to bring together actors from multiple systems that do not historically interact in order to increase access to health care for an underserved and at-risk population. This was a pilot study designed to demonstrate proof of concept and the results indicate that the concept appears viable. Replications are needed to confirm these findings with other healthcare and justice system agencies and in other locations.

## Data Availability

The datasets generated and/or analyzed during the current study are not publicly available due to ongoing analyses by the authors, but are available from the corresponding author on reasonable request.
